# Clinicopathologic Analysis of Localized Nasal/Paranasal Diffuse Large B-Cell Lymphoma

**DOI:** 10.1371/journal.pone.0057677

**Published:** 2013-02-28

**Authors:** Hiroko Toda, Yasuharu Sato, Katsuyoshi Takata, Yorihisa Orita, Naoko Asano, Tadashi Yoshino

**Affiliations:** 1 Department of Pathology, Okayama University Graduate School of Medicine, Dentistry, and Pharmaceutical Sciences, Okayama, Japan; 2 Department of Otolaryngology, Head and Neck Surgery, Okayama University Graduate School of Medicine, Dentistry, and Pharmaceutical Sciences, Okayama, Japan; 3 Department of Clinical Laboratory, Nagoya University Hospital, Nagoya, Japan; The University of North Carolina at Chapel Hill, United States of America

## Abstract

Diffuse large B-cell lymphoma (DLBCL) comprises 2 molecularly distinct subgroups of non-germinal center B-cell-like (non-GCB) and germinal center B-cell-like (GCB) DLBCLs, with the former showing relatively poor prognosis. In the present study, we analyzed the clinicopathological features of 39 patients with localized nasal/paranasal DLBCL. Immunohistochemistry-based subclassification revealed that 11 patients (28%) were of the GCB-type according to Hans’ algorithm and 11 (28%) were of the GCB-type according to Choi’s algorithm. According to both Hans’ and Choi’s algorithms, the non-GCB type was predominant. Nevertheless, prognosis was good. Overall survival did not differ significantly between the GCB and non-GCB subgroups (Hans’ algorithm: *p* = 0.57, Choi’s algorithm: *p* = 0.99). Furthermore, the prognosis of localized nasal/paranasal DLBCL was better than that of other localized extranodal DLBCLs. The prognosis of extranodal DLBCL is usually considered poorer than that of nodal DLBCL. However, in our study, no difference was noted between patients with localized nasal/paranasal DLBCL and patients with localized nodal DLBCL. In conclusion, although the non-GCB subtype is thought to show poor prognosis, in our study, the prognosis for localized nasal/paranasal DLBCL patients was good irrespective of subclassification.

## Introduction

Although heterogeneous in nature, diffuse large B-cell lymphoma (DLBCL) can be classified into 2 distinct subtypes on the basis of genetic profiling: the germinal center B-cell-like (GCB) phenotype and the non-germinal center B-cell-like (non-GCB) phenotype [1], [2], [3]. Notably, patients belonging to the former group have a better prognosis than those belonging to the latter group. Hans et al. reported that these DLBCL subtypes can be easily distinguished on the basis of immunohistological staining for CD10, BCL6, and MUM1 proteins [2]. Later, Choi et al. added 2 new antibodies, FoxP1 and GCET1 [4], and Choi’s algorithm is reported to achieve better prognostic classification than Hans’ algorithm [5]. Extranodal non-GCB-like DLBCL is generally characterized by poor prognosis regardless of its localized disease, but localized primary non-tonsillar oral DLBCL exhibits favorable prognosis even in cases of the non-GCB subtype [6]. Nasal/paranasal DLBCL is uncommon, and the GCB and non-GCB subtypes of this disease have not yet been examined. In this study, we aimed to clarify the clinicopathological features of localized nasal/paranasal DLBCL.

## Materials and Methods

### Patients

We selected 39 Japanese patients diagnosed with localized nasal/paranasal DLBCL between 1995 and 2010 and reviewed our institution’s pathology department database to obtain the medical records of these patients. We only evaluated localized lymphomas, because the primary sites of advanced lymphomas are difficult to determine. All 39 cases were diagnosed as primary extranodal DLBCLs. Patients were defined as having extranodal DLBCL when the disease was confined to one or more extranodal sites and showed no (or only minor) nodal involvement after the staging procedures [7], [8]. This group of patients was then compared with 39 patients with localized nodal DLBCLs diagnosed at our institution [9]. The samples and the medical records (clinical history, treatment and survival data) used in our study was approved by the Institute Review Board (IRB) at Okayama University. Written informed consent was waived by our institutional review board, since our study was limited to the use of excess human tissue samples and medical records.

### Histological Examination and Immunohistochemistry

Surgically resected or biopsied specimens of localized nasal/paranasal DLBCLs were fixed in 10% formaldehyde and embedded in paraffin. Serial sections (4 µm) were cut from each paraffin-embedded tissue block, and several of these sections were stained with hematoxylin. To subclassify the GCB- or non-GCB- type of DLBCL, immunohistochemistry was performed on formalin-fixed, paraffin-embedded sections using an automated Bond Max stainer (Leica Biosystems, Melbourne, Australia). The primary antibodies used were as follows: CD20 (L26, 1∶200; Novocastra, Newcastle-upon-Tyne, UK), CD3 epsilon (LN10, 1∶200; Novocastra), BCL6 (D8, 1∶100; SantaCruz), CD5 (4C7, 1∶100; Novocastra), GCET1 (RAM341, 1∶100; Abcam), CD10 (56C6, 1∶50; Novocastra), MUM1 (MUM1p, 1∶50; Dako), FoxP1 (JC12, 1∶500; LifeSpan Biosciences, Seattle, USA), and Ki-67 (MIB-1, 1∶5000; Novocastra). For each section, 10 high-power fields were recorded, quantitated, and averaged to calculate the estimated percentage of positively immunostained cells. Negativity for CD10, BCL6, and MUM1 was defined as <30% positively stained tumor cells, and positivity was defined as >30% positively stained tumor cells. As an exception, for Choi’s algorithm, negativity for MUM1 was defined as <80% positively stained tumor cells, and positivity, as >80% positively stained tumor cells. Negativity for GCET1 and FoxP1 staining was defined as <80% positively stained tumor cells, and positivity, as >80% positively stained tumor cells. Ki-67 immunoreactivity was evaluated semi-quantitatively by using the average estimated percentage of positive cells in the 10 recorded high-power fields [4].

### Statistical Analysis

All data were analyzed using STATA software (version 9.0; Stata Corp., College Station, TX, USA). Actuarial overall survival curves were calculated using the Kaplan-Meier method, and differences were examined using the log-rank test to determine significant prognostic factors [10]. Overall survival was defined as the time from diagnosis to death from any cause or to the last follow-up visit.

## Results

### Characteristics of the Nasal/Paranasal DLBCL Cases

**Figure 1:**
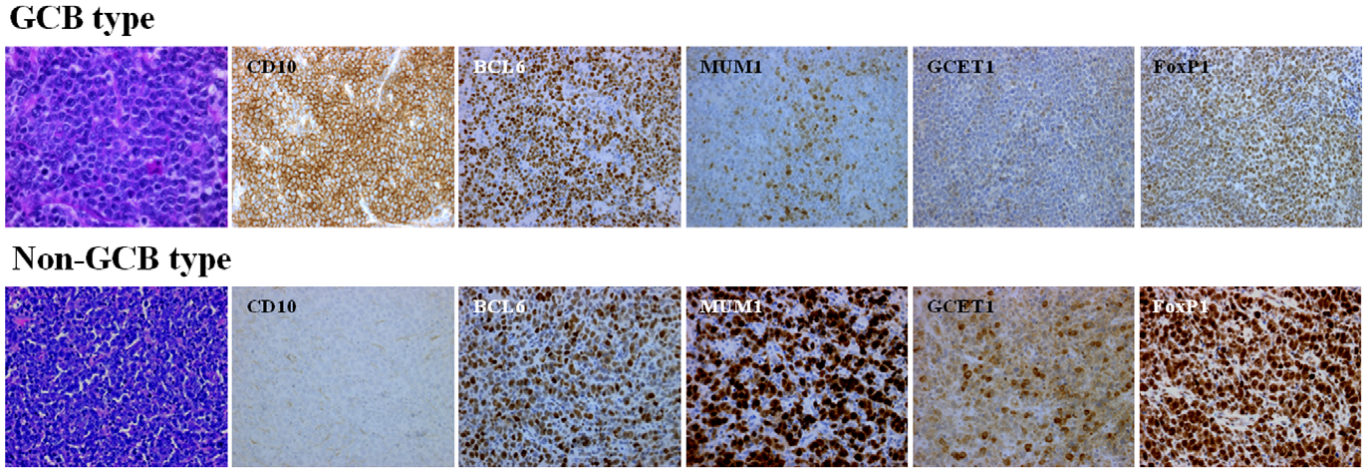
Histological and immunohistochemical features. Diffuse infiltration and proliferation of large lymphoma cells (Hematoxylin–eosin staining).

Table 1 and Table 2 summarize the characteristics of the localized nasal/paranasal DLBCL patients. The median age of the 39 patients was 76 years (range, 33–98 years). The patient population comprised 21 men and 18 women. According to Choi’s algorithm, 11 of the 39 patients (28%) were of the GCB- type and 28 (72%) were of the non-GCB- type. According to Hans’ algorithm, 11 (28%) were of the GCB- type and 28 of the 39 patients (72%) were of the non-GCB type (Table 3). According to the Ann Arbor classification, 33 patients were at clinical stage IE and 6 were at stage IIE. According to the International Prognostic Index**,** 4 patients were at low- intermediate risk and 13 were at low risk. Histologically, all cases were classified as DLBCL (Fig. 1). All patients were newly presenting with no prior treatment history.

**Table 1:** Clinical data for 39 localized nasal/paranasal DLBCLs.

Patient no.	Age (years)	Sex	Primary site	IPI	LDH>normal values	Tumor size (maximum diameter; cm)	CS	Treatment	Relapse	Follow-up period	Follow-up
										(months)	status
1	93	M	left nasal cavity	L or LI	No	NA	IE	RT	NA	3	DOAD
2	72	F	right nasal cavity	LI or HI	Yes	5	IE	R-THP-COP	NA	NA	NA
3	57	M	left nasal cavity/paranasal sinus	L	No	NA	IE	CHOP+RT	−	48	Alive,FOD
4	60	F	left nasal cavity	L	No	NA	IE	R-CHOP+RT	−	34	Alive,FOD
5	94	F	left nasal cavity	L or LI or HI	NA	NA	IE	NA	NA	NA	NA
6	69	F	left nasal cavity/paranasal sinuses	L or LI or HI	No	3.7	IE	CHOP+RT	−	NA	NA
7	76	M	left nasal cavity/paranasal sinuses	L	No	4	IE	Epi-COP+RT	−	83	Alive,FOD
8	83	F	right nasal cavity/paranasal sinuses	LI	Yes	6	IE	Epi-COP	−	4	DOD
9	56	F	right nasal cavity/paranasal sinus/lymph nodes	L	No	3.5	IIE	CHOP+RT	−	101	Alive,FOD
10	85	F	right nasal cavity	HI	Yes	3.8	IE	RT	+	13	DOAD
11	73	F	left nasal cavity/lymph nodes/paranasal sinuses	L	No	NA	IIE	CHOP+RT	NA	7	AWD
12	74	M	left nasal cavity	LI or HI	Yes	NA	IE	CHOP+RT	+	50	Alive,FOD
13	89	F	left nasal cavity/paranasal sinus	LI or HI	Yes	NA	IE	NA	NA	NA	NA
14	81	F	left nasal cavity	L or LI	No	NA	IE	R-THPCOP+RT	−	17	Alive,FOD
15	67	M	paranasal sinus	L or LI or HI	NA	NA	IE	CHOP	−	125	Alive,FOD
16	64	F	left nasal cavity/paranasal sinus	L	No	NA	IE	RT+chemotherapy	NA	NA	NA
17	89	F	left nasal cavity/paranasal sinus/lymph node	L or LI	NA	NA	IIE	NA	NA	NA	NA
18	84	F	right nasal cavity/pharynx	L or LI or HI	NA	NA	IE	NA	NA	NA	NA
19	77	M	left nasal cavity/paranasal sinus	L or LI	NA	NA	IE	NA	NA	NA	NA
20	75	M	right nasal cavity/paranasal sinus	L	No	NA	IE	NA	−	19	DOAD
21	77	M	right nasal cavity/paranasal sinus	LI or HI	Yes	5.5	IE	R-THP-COP	+	17	DOD
22	79	M	left nasal cavity	L or LI	No	3	IE	chemo+RT	−	11	Alive,FOD
23	71	F	left nasal cavity/lymph nodes	L or LI	No	2.5	IIE	chemotherapy	−	35	Alive,FOD
24	98	M	right nasal cavity	LI or HI	Yes	2	IE	untreatment	NA	0	DOAD
25	80	M	bilateral nasal cavities/paranasal sinus/lymph nodes	L	No	3.7	IIE	R-THP-COP	+	36	AWD
26	65	M	nasal cavity	L	No	NA	IE	R-CHOP+RT	−	30	Alive,FOD
27	68	M	right paranasal sinus/lymph nodes	LI	No	2.4	IIE	R-CHOP	−	20	Alive,FOD
28	33	M	left nasal cavity	L	No	3	IE	NA	NA	NA	NA
29	77	F	right paranasal sinuses	L or LI	NA	NA	IE	R-CHOP+MTX	+	23	Alive,FOD
30	80	M	right nasal cavity/right paranasal sinuses	LI	No	2	IE	untreatment	NA	3	AWD
31	78	F	right nasal cavity	L or LI or HI	NA	2.5	IE	NA	NA	NA	NA
32	72	M	left paranasal sinuses	L or LI	No	5	IE	NA	NA	NA	NA
33	77	M	right nasal cavity	L or LI or HI	NA	NA	IE	R-THP-COP+RT	+	23	AWD
34	76	M	left nasal cavity/paranasal sinuses	L	No	4	IE	R-THP-COP+RT	−	9	Alive,FOD
35	78	F	right nasal cavity	L or LI	No	NA	IE	untreatment	NA	2	AWD
36	68	M	left nasal cavity/paranasal sinuses	L	No	4	IE	R-CHOP	NA	1	DOD
37	74	F	left nasal cavity	L or LI	No	NA	IE	R-CHOP+RT	−	12	Alive,FOD
38	57	M	right paranasal sinuses	L	No	3	IE	NA	NA	27	Alive,FOD
39	83	M	left nasal cavity/paranasal sinus	LI	No	3.6	IE	R-THP-COP+RT	+	48	Alive,FOD

CHOP, cyclophosphamide, adriamycin, vincristine, prednisolone; CS, clinical stage; F, female; FOD, free of disease; AWD, alive with disease; DOD, dead of disease; DOAD, dead of another disease; IPI, International Prognostic Index; L, low; LDH, lactate dehydrogenase; LI, low–intermediate;

M, male; NA, not available; MTX, methotrexate; R-, with rituximab; RT, radiation; THP-COP, pirarubicin, cyclophosphamide, vincristine, prednisolone; Epi-COP, epirubicin, cyclophosphamide, vincristine, prednisolone.

**Table 2:** Clinical characteristics of patients with localized nasal/paranasal DLBCL.

Characteristic	No. of patients (%)
Sex	
Male	21(54)
Female	18(46)
Age (y), median (range)	76 (33–98)
Ann Arbor stage	
I	33(85)
II	6(15)
LDH	
NormalElevated	24(77)7(23)
PS	
0–1	15(79)
2 or more	4(21)
IPI	
L-LI	27(96)
HI-H	1(4)
Treatment	
chemotherapy	8(32)
chemotherapy+RT	15(60)
RT alone	2(8)
Complete Remission	
yes	22(88)
no	3(12)

**Table 3:** Clinical and phenotypic characteristics of patients with GCB-type and non-GCB-type DLBCL.

	Total (n = 39)	Hans’ algorithm		*P*	Choi’ algorithm		*p*
		GCB (n = 11)	non-GCB (n = 28)		GCB (n = 11)	non-GCB (n = 28)	
**Sex (male/female)**	21/18	6/5	15/13	0.96	6/5	14/14	0.80
**Age (y), median (range)**	76 (33–98)	75 (57–98)	77 (33–94)	0.61	77 (57–98)	76 (33–94)	0.98
** Age >60**	34/39 (87%)	10/11 (91%)	24/28 (86%)	0.66	10/11 (91%)	24/28 (86%)	0.66
** PS >1**	4/19 (21%)	0/5 (0%)	4/14 (29%)	0.18	0/5 (0%)	4/14 (29%)	0.18
** B symptoms**	1/29 (3%)	1/8 (13%)	0/21 (0%)	0.099	1/8 (13%)	0/21 (0%)	0.099
** LDH >normal**	7/31 (23%)	2/8 (25%)	5/23 (22%)	0.85	2/9 (22%)	5/22 (23%)	0.98
**Median survival** **(months)**	23 (1–125+)	35 (11–125+)	20 (1–101+)	0.57	35 (11–48+)	23 (1–125+)	0.99
**Immunophenotype**							
** CD10**	8/39 (21%)	8/11 (73%)	0/28 (0%)	<0.0001	6/11 (55%)	2/28 (7%)	0.00097
** MUM1(Hans)**	28/39 (72%)	5/11 (45%)	23/28 (82%)	0.022			
** MUM1(Choi)**	24/39 (62%)				3/11 (27%)	21/28 (75%)	0.0058
** BCL6**	25/39 (64%)	9/11 (82%)	16/28 (57%)	0.15	10/11 (91%)	15/28 (54%)	0.029
** FOXP1**	29/39 (74%)				6/11 (55%)	23/28 (82%)	0.076
** GCET1**	12/39 (31%)				3/11 (27%)	9/28 (32%)	0.77

Abbreviations: GCB, germinal center B-cell; PS, performance status; BM, bone marrow; LDH, lactate dehydrogenase; FOXP1, forehead box protein 1; GCET1, germinal center B-cell expressed transcript 1.

### Phenotypic Features of the Localized Nasal/Paranasal DLBCL Cases

**Figure 2:**
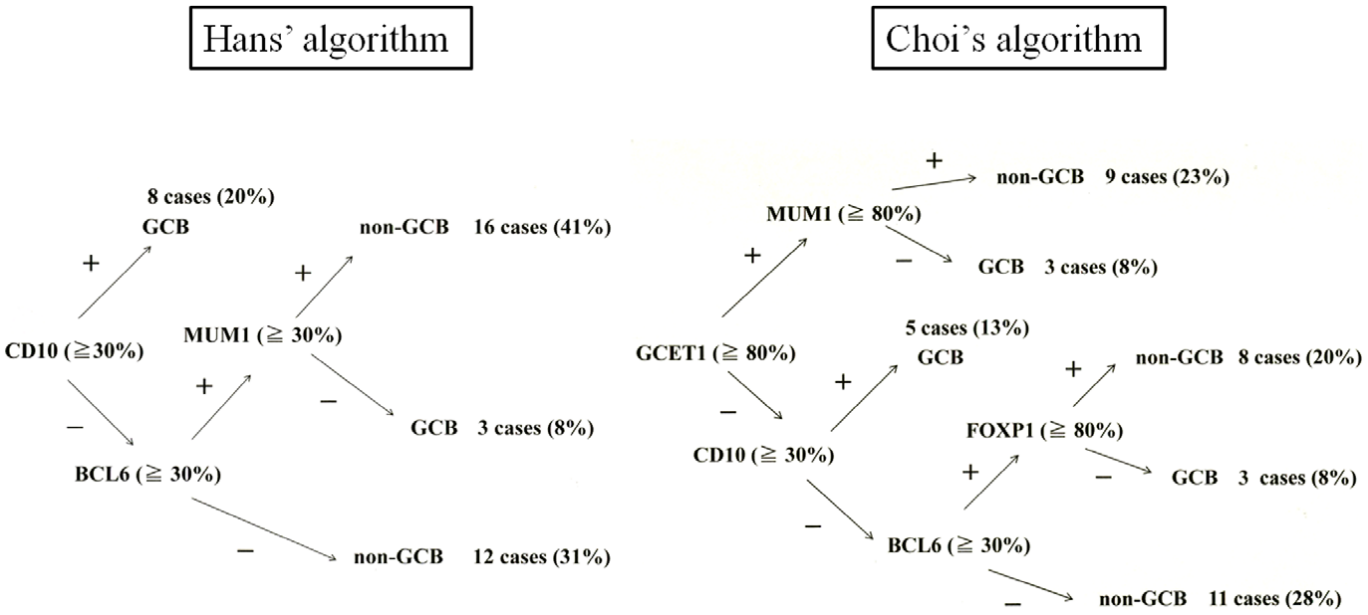
Distribution of GCB and non-GCB type according to Hans et al. and Choi et al.

**Figure 3:**
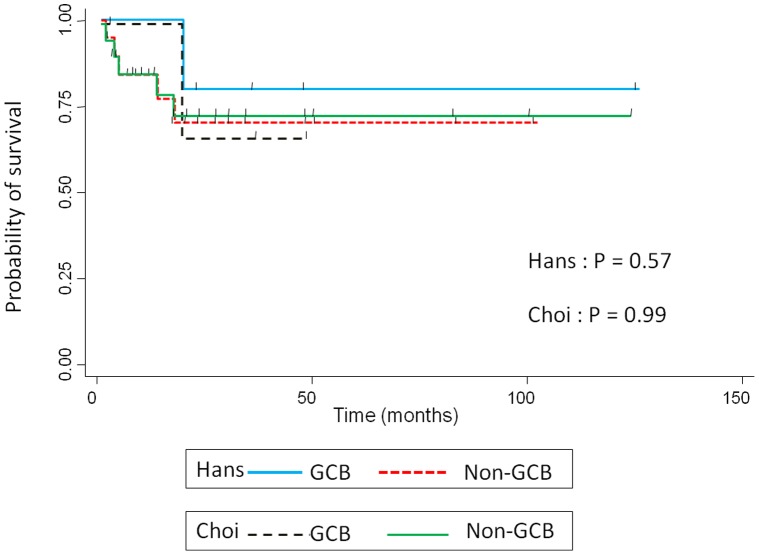
Kaplan–Meier plot showing overall survival for patients with localized nasal/paranasal DLBCL.

Table 4 summarizes the phenotypic features of the localized nasal/paranasal DLBCL patients. The B-cell immunophenotype of the lymphomas was confirmed by immunoreactivity with antibodies to CD20 in 39 cases. Although no cases were positive for CD5, 8 (21%) were positive for CD10 and 25 (64%) were positive for BCL6. For MUM1 staining, 28 cases (72%) were positive according to Hans’ algorithm and 24 cases (62%) were positive according to Choi’s algorithm. Furthermore, 29 cases (74%) were positive for FoxP1 and 12 (31%) were positive for GCET1. Of the 11 cases (28%) classified as GCB- type according to Hans’ algorithm, 8 were CD10-positive cases (20%), and 3 were CD10-negative, BCL6-positive, MUM1-negative cases (8%). Of the 11 (28%) classified as GCB- type according to Choi’s algorithm, 3 were GCET1-positive, MUM1-negative cases (8%); 5 were GCET1-negative, CD10-positive cases (13%); and 3 GCET1-negative, CD10-negative, BCL6-positive, FoxP1-negative cases (8%). Of the 28 cases (72%) classified as the non-GCB- type according to Hans’ algorithm, 12 were CD10-negative, BCL6-negative cases (31%) and 16 were CD10-negative, BCL6-positive, MUM1-positive cases (41%). Of the 28 cases (72%) classified as the non-GCB- type according to Choi’s algorithm, 9 were GCET1-positive, MUM1-positive cases (23%); 8 were GCET1-negative, CD10-negative, BCL6-positive, FoxP1-positive cases (20%); and 11 were GCET1-negative, CD10-negative, BCL6-negative cases (28%) (Fig. 2). The non-GCB- type was dominant according to both algorithms, but the prognosis for these cases was good. Overall survival did not differ significantly between the non-GCB type and GCB type groups (*p = *0.57, Hans’ algorithm, *p = *0.99, Choi’s algorithm) (Fig. 3).

**Table 4:** Immunohistochemical findings of localized nasal/paranasal DLBCLs.

Patient no.	CD3	CD5	CD10	CD20	Ki-67 labeling (%)	MUM1 (Hans)	MUM1 (Choi)	BCL6	EBER	FOXP1	GCET1	subtype(Hans)	subtype(Choi)
1	−	−	−	+	21	U.D.	−	−	−	+	−	Non-GCB	Non-GCB
2	−	−	p+	+	43	−	−	+	−	+	−	GCB	GCB
3	−	−	+	+	43	−	−	+	−	−	−	GCB	GCB
4	−	−	−	+	80	+	+	−	−	+	−	Non-GCB	Non-GCB
5	−	−	−	+	59	+	+	−	−	+	−	Non-GCB	Non-GCB
6	−	−	−	+	54	+	+	+	−	+	−	Non-GCB	Non-GCB
7	−	−	−	+	71	+	+	+	−	+	−	Non-GCB	Non-GCB
8	−	−	−	+	82	+	+	−	−	+	−	Non-GCB	Non-GCB
9	−	−	−	+	71	−	−	−	−	+	−	Non-GCB	Non-GCB
10	−	−	−	+	61	+	+	+	−	−	+	Non-GCB	Non-GCB
11	−	−	−	+	50	+	+	+	−	+	−	Non-GCB	Non-GCB
12	−	−	−	+	34	+	+	+	−	−	+	Non-GCB	Non-GCB
13	−	−	−	+	55	+	+	+	−	+	+	Non-GCB	Non-GCB
14	−	−	−	+	70	+	+	+	−	+	+	Non-GCB	Non-GCB
15	−	−	+	+	64	+	+	−	−	−	+	GCB	Non-GCB
16	−	−	−	+	33	−	−	+	−	−	+	GCB	GCB
17	−	−	−	+	61	+	+	−	−	+	+	Non-GCB	Non-GCB
18	−	−	−	+	72	+	+	−	−	−	−	Non-GCB	Non-GCB
19	−	−	−	+	63	+	+	+	−	−	−	Non-GCB	GCB
20	−	−	−	+	67	−	−	+	−	−	−	GCB	GCB
21	−	−	−	+	80	+	+	−	−	−	−	Non-GCB	Non-GCB
22	−	−	+	+	80	+	+	−	−	+	−	GCB	GCB
23	−	−	+	+	72	−	−	+	−	+	+	GCB	GCB
24	−	−	+	+	90	+	+	+	−	+	−	GCB	GCB
25	−	−	−	+	73	−	−	+	−	+	+	GCB	GCB
26	−	−	−	+	81	+	+	+	−	+	−	Non-GCB	Non-GCB
27	−	−	−	+	52	+	+	+	−	+	−	Non-GCB	Non-GCB
28	−	−	−	+	72	+	−	−	−	+	−	Non-GCB	Non-GCB
29	−	−	+	+	97	+	+	+	−	+	+	GCB	Non-GCB
30	−	−	−	+	90	−	−	−	−	+	−	Non-GCB	Non-GCB
31	−	−	+	+	77	+	−	+	−	+	−	GCB	GCB
32	−	−	−	+	49	−	−	−	−	+	−	Non-GCB	Non-GCB
33	−	−	−	+	88	+	+	+	−	+	+	Non-GCB	Non-GCB
34	−	−	−	+	54	+	+	+	−	+	−	Non-GCB	Non-GCB
35	−	−	−	+	58	+	−	+	−	−	−	Non-GCB	GCB
36	−	−	−	+	92	+	−	+	−	+	−	Non-GCB	Non-GCB
37	−	−	−	+	61	−	+	+	−	+	−	Non-GCB	Non-GCB
38	−	−	−	+	90	+	−	−	−	+	−	Non-GCB	Non-GCB
39	−	−	−	+	83	+	+	+	−	+	+	Non-GCB	Non-GCB

EBER, Epstein–Barr virus-encoded small RNA; GCB, germinal center B-cell; FOXP1, forehead box protein 1; GCET1; germinal center B-cell expressed transcript 1.

### Therapeutic Response and Outcome

Follow-up clinical data were available for 28 patients. The duration of follow-up ranged from 1 to 125 months (mean, 29 months). Fifteen patients were initially treated with chemotherapy plus irradiation, 9 were treated with chemotherapy alone, and 2 were treated with irradiation alone. Twenty-two patients achieved complete remission. Although 7 patients relapsed, 3 of these patients achieved complete remission following alternative chemotherapy. Salvage treatments for the 7 relapsed patients were R-MFP (methotrexate, fluorouracil, low dose cisplatin, and rituximab), R-THP-COP (cyclophosphamide, vincristine, prednisolone, pirarubicin, and rituximab), R-MTX (methotrexate and rituximab), and CHASER (cyclophosphamide, high dose cytarabine, dexamethasone, etoposide, and rituximab) plus radiation. At the time of reporting, 16 patients were disease- free and 3 patients had died of the disease.

### Comparison of the Clinicopathological Characteristics of Localized Nasal/Paranasal DLBCL and Localized Nodal DLBCL

**Figure 4:**
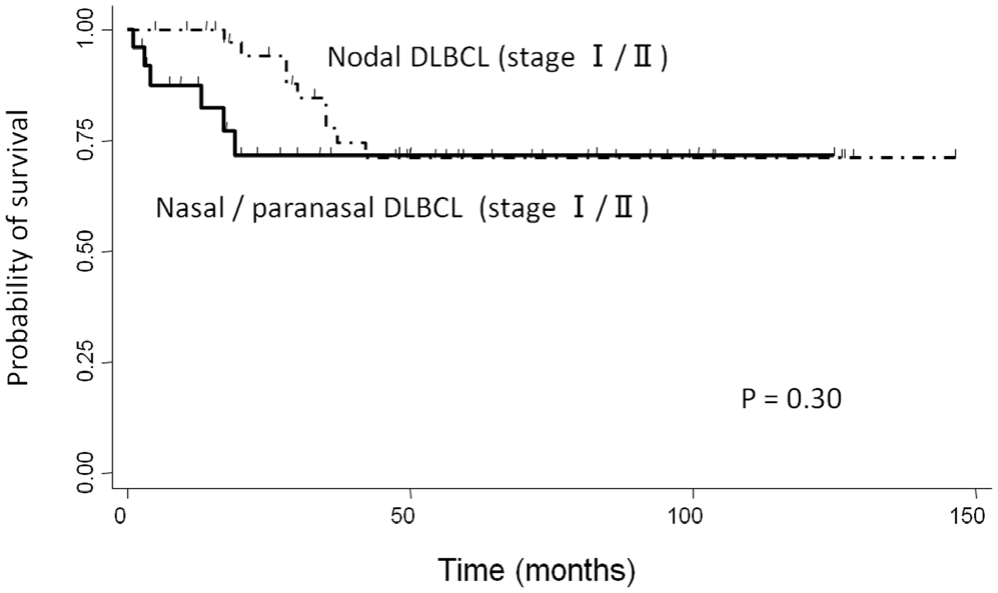
Comparison of overall survival between localized nasal/paranasal DLBCL and localized nodal DLBCL.

The clinicopathological characteristics of nasal/paranasal DLBCL and localized nodal DLBCL are summarized in Table 5. Nasal/paranasal DLBCL patients showed good prognosis. The slight difference in the overall survival between these patients and patients with localized nodal DLBCL was not significant (*p = *0.30) (Fig. 4). Moreover, analysis using the χ2-test revealed a significant difference between the 2 groups with regard to age distribution and immunophenotype. Localized nasal/paranasal DLBCL patients were more likely to be more than 60 years old than localized nodal DLBCL patients (*p = *0.018). In addition, localized nasal/paranasal DLBCL patients showed significantly higher positivity for MUM1 than localized nodal DLBCL patients according to Choi’s algorithm (*p = *0.00023, χ2-test).

**Table 5:** Clinical and phenotypic characteristics of patients with localized nasal/paranasal DLBCL and localized nodal DLBCL.

	localized nasal/paranasal DLBCL	localized nodal DLBCL	*p*
	Total (n = 39)	Total (n = 39)	
**Sex (male/female)**	21/18	23/16	0.65
**Age (y), median (range)**	76 (33–98)	70 (33–79)	0.0010
** Age >60**	34/39 (87%)	25/39 (64%)	0.018
** IPI : L-LI**	27/28 (96%)	38/39 (97%)	0.81
** Relapse**	7/22 (32%)	16/39 (41%)	0.48
** LDH >normal**	7/31 (23%)	7/39 (18%)	0.63
**Median survival (months)**	23 (1–125+)	49 (4–146+)	0.30
**Immunophenotype**			
** CD10**	8/39 (21%)	16/39 (41%)	0.050
** MUM1(Hans)**	28/39 (72%)	28/39 (72%)	1.0
** MUM1(Choi)**	24/39 (62%)	8/39 (21%)	0.00023
** BCL6**	25/39 (64%)	31/39 (79%)	0.13
** FOXP1**	29/39 (74%)	32/39 (82%)	0.41
** GCET1**	12/39 (31%)	17/39 (44%)	0.24

Abbreviations: GCB, germinal center B-cell; PS, performance status; LDH, lactate dehydrogenase.

## Discussion

**Figure 5:**
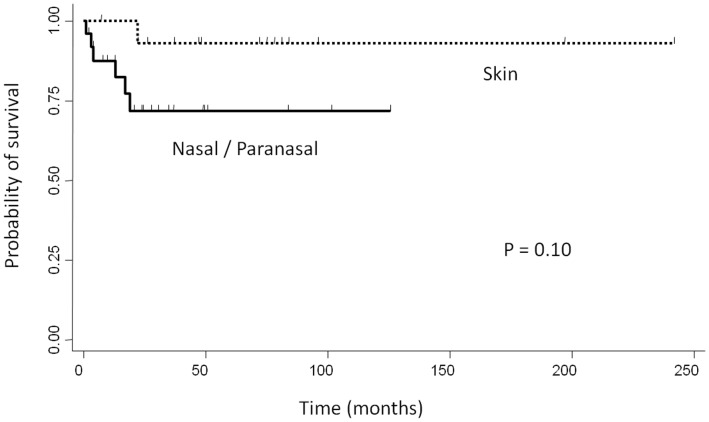
Comparison of overall survival between localized nasal/paranasal DLBCL and localized skin DLBCL.

**Figure 6:**
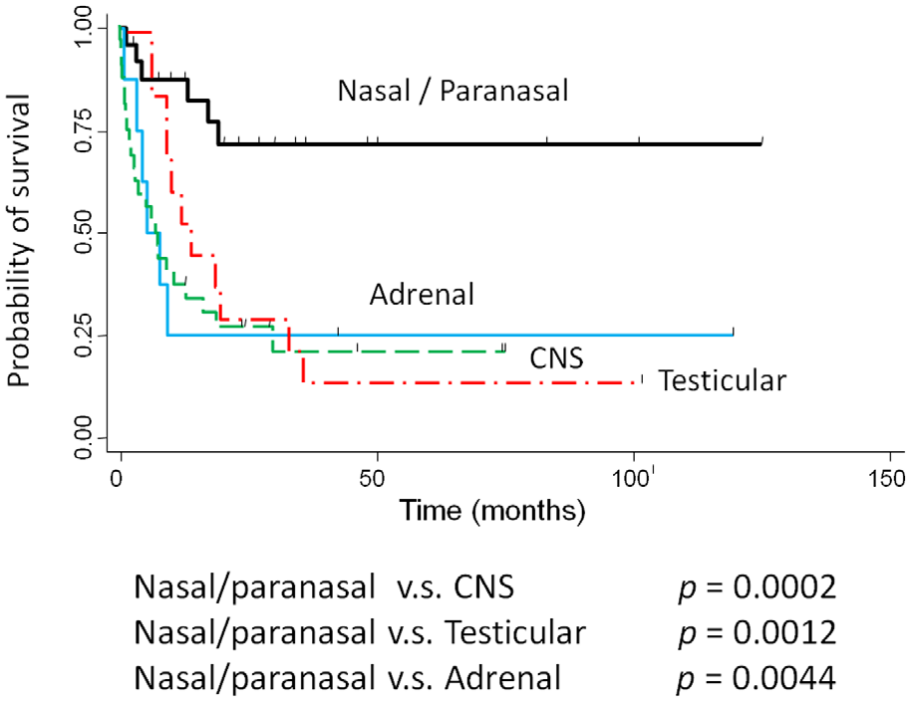
Comparison of overall survival between localized nasal/paranasal DLBCL and localized adrenal, CNS, and testicular DLBCLs.

DLBCL is the most frequent and aggressive lymphoma, representing a heterogeneous group that includes *de novo* large B-cell lymphomas, as well as transformed lymphomas from follicular or mucosa-associated lymphoid tissue lymphomas [11]. Recent studies have demonstrated that DLBCL can be further subclassified into 2 major prognostic categories according to Hans et al.: the GCB- type and the non-GCB- type [1], [2]. However, Hans’ algorithm has been superseded by a new algorithm devised by Choi et al., and results obtained using Choi’s algorithm closely correlate with those of gene expression profiling for predicting prognosis [5]. In general, the non-GCB- type of DLBCL is associated with a significantly poorer prognosis than the GCB- type [1]; however, it has recently been established that this may not be true for extranodal DLBCL. Patients with localized primary non-tonsillar oral DLBCL presented with a favorable clinical course despite having the non-GCB- type [6]. Similarly, in our study, the non-GCB- type of localized nasal/paranasal DLBCL was the dominant type following subclassification according to both algorithms, but the prognosis of these patients was good. Moreover, the prognosis of localized nasal/paranasal DLBCL was as good as that of primary cutaneous DLBCL [12] (*p = *0.10) (Fig. 5) and was statistically better than that of other localized extranodal DLBCLs (CNS [13], testis [14], and adrenal gland [15]) **(** *p = *0.0002, *p = *0.0012, and *p = *0.0044, respectively) (Fig. 6). Generally, extranodal non-GCB-like DLBCLs are characterized by poor prognosis, and the incidence of non-GCB- type DLBCLs among extranodal DLBCLs is 83–100%, although this value differs according to the organ of manifestation [16], [17], [18], [19]. According to previous reports, DLBCLs of the central nervous system [16], breast [17], stomach [20], leg type [21], testis [18], and intravascular type [19] are predominantly of the non-GCB- type, an observation consistent with the finding in our study of localized nasal/paranasal DLBCL cases. However, patients with CNS, breast, and testicular DLBCL exhibit poor prognosis, regardless of the localized disease [16], [17], [18], [22]. As shown for primary cutaneous B-cell lymphoma, findings of genes expression analysis suggest that primary non-leg-type cutaneous DLBCL and primary cutaneous DLBCL, leg type have expression profiles similar to those of GCB- type and non-GCB- type DLBCLs, respectively [23]. Therefore, primary non-leg-type cutaneous DLBCL is predominantly associated with an excellent prognosis [12], [24]. According to the recent World Health Organization (WHO) classification, subsets of DLBCLs arising in peculiar extranodal sites have been categorized as distinct disease subgroups (primary DLBCLs of the CNS, primary cutaneous DLBCLs, leg-type) or as distinct disease entities (primary mediastinal large B-cell lymphoma), on the basis of specific clinical and/or pathologic features [25], [26]. When the cases in our study are included, extranodal disease is common among DLBCL patients [27]. It is thought that there are important clinical differences between nodal and extranodal DLBCL and that the most reliable distinction can be made in patients with stage I disease. For these patients, extranodal DLBCL is independently associated with poor survival [27]. Therefore, we also compared the clinicopathological profiles of localized nasal/paranasal DLBCLs with localized nodal DLBCLs. This analysis showed that localized nasal/paranasal DLBCL was associated with good prognosis and no difference was noted in the prognosis compared with localized nodal DLBCL. In recent years, the use of rituximab has improved the prognosis of DLBCL patients, and CHOP (cyclophosphamide, adriamycin, vincristine, and prednisolone) therapy combined with rituximab (R-CHOP) is currently a standard chemotherapy for DLBCL [28]. In our study, no significant difference was noted in the number of patients treated with rituximab between localized nasal/paranasal DLBCL and localized nodal DLBCL patients (*p = *0.24). Therefore, the prognosis of localized nasal/paranasal DLBCLs was favorable regardless of treatment with rituximab. In conclusion, the prognosis of localized nasal/paranasal DLBCL patients was good irrespective of the disease subclassification, although the non-GCB- type of DLBCLs are usually thought to be associated with a poor prognosis.
